# Multilevel legal approaches to obesity prevention: A conceptual and methodological toolkit

**DOI:** 10.1371/journal.pone.0220971

**Published:** 2019-10-01

**Authors:** Sara E. Abiola, Michelle M. Mello

**Affiliations:** 1 Department of Health Policy & Management, Columbia Mailman School of Public Health, New York, New York, United States of America; 2 Department of Health Research and Policy, Stanford School of Medicine, Stanford Law School, Palo Alto, California, United States of America; Massachusetts Department of Public Health, UNITED STATES

## Abstract

**Introduction:**

State lawmakers have explored numerous policy alternatives to reduce overweight and obesity. Evaluating effects of these laws is important but presents substantial methodological challenges. We present a conceptual framework that allows for classification of obesity prevention laws based on ecological level of influence and the underlying legal mechanism involved to guide analysis of the relationship between a substantial range of obesity prevention laws and BMI.

**Methods:**

Obesity prevention laws (OPLs) for all 50 states and DC were obtained via primary legal research using the LexisNexis Advanced Legislative Services (ALS) database. For legal provisions that met inclusion criteria, reviewers abstracted information on bill state, citation, passage and effective dates, target population, and obesity prevention mechanism. Laws were categorized by ecological level of influence on weight-related behaviors and the legal mechanism utilized to change behavioral determinants of BMI.

**Results:**

Laws designed to increase community-level opportunities for physical activity were the most frequently enacted OPL while laws designed to alter nutrition standards for school meals or competitive foods were comparatively less common, appearing in only 16% and 34% of states, respectively.

**Conclusion:**

Prior studies of obesity policies have focused on specific interventions. We identified and categorized state-level laws that operate at all ecological levels and found that laws passed during the initial burst of lawmaking were largely confined to measures aimed at increasing opportunities for physical activity. Creating public spaces for recreation is an important step to promoting healthier lifestyles to reduce obesity risk; more comprehensive, multilevel legal approaches should also be pursued.

## Introduction

Over the past four decades, the prevalence of childhood overweight has more than doubled for children ages 2 to 11 and rates have more than tripled for those aged 12 to 19 [1]. Thirty-two percent of children and adolescents are overweight or obese and obesity and severe obesity continues to increase significantly among children aged 2 to 5 and among adolescent females [2]. The economic, physical, psychological, and social consequences of overweight and obesity are well documented [3–7].

U.S. states have broad authority to enact laws to address obesity and its consequences using the “police power”—the constitutional power of state governments to promote the public’s health, safety, and welfare [8]. State legislatures have exercised this power extensively with the aim of preventing obesity among children and adolescents [9, 10]. State laws range from establishing nutrition education standards to increasing standards for physical activity during the school day. Several national organizations and research groups have compiled extensive catalogs of these legal approaches to overweight and obesity [11–16]. Moreover, recent studies examine the relationship between particular state-level obesity prevention laws and BMI in adolescent or young adult populations [17–22]. While this work has been evolving, public health law researchers have developed methods for systematically and reliably gathering, coding, and modeling the effects of laws on population health [23–26]. These methods have not yet been applied to a comprehensive set of obesity prevention laws.

In this article, we draw on existing work in the social determinants of health and gold-standard methods in the field of public health law research (PHLR) to present a conceptual framework linking laws to obesity-related health outcomes and a protocol for the collection and coding of obesity prevention laws. For our purposes, obesity prevention laws (OPLs) are defined as state statutes that have the express purpose of preventing or reducing obesity, as well as statutes designed with the primary goal of promoting behaviors known to be associated with obesity prevention or reduction, such as healthy eating and physical activity.

## Methods

### Public health laws and population health

The pathway from lawmaking to population health includes several intermediate steps, each of which can be examined empirically. PHLR can examine the factors that influence policy adoption, implementation or enforcement of laws, effect of laws on environments and health behaviors, effect of laws on environments that encourage behavior change, and ultimately the relationship between environmental and behavioral change that lead to shifts in population health. Our focus here is primarily on the effect of law on behaviors related to overweight and obesity. The goal is to determine the extent to which laws reduce youth body mass index (BMI) either by directly influencing these behaviors or by effecting environmental changes that in turn shape behaviors.

### Obesity prevention laws and ecological models of obesity

**Fig 1:**
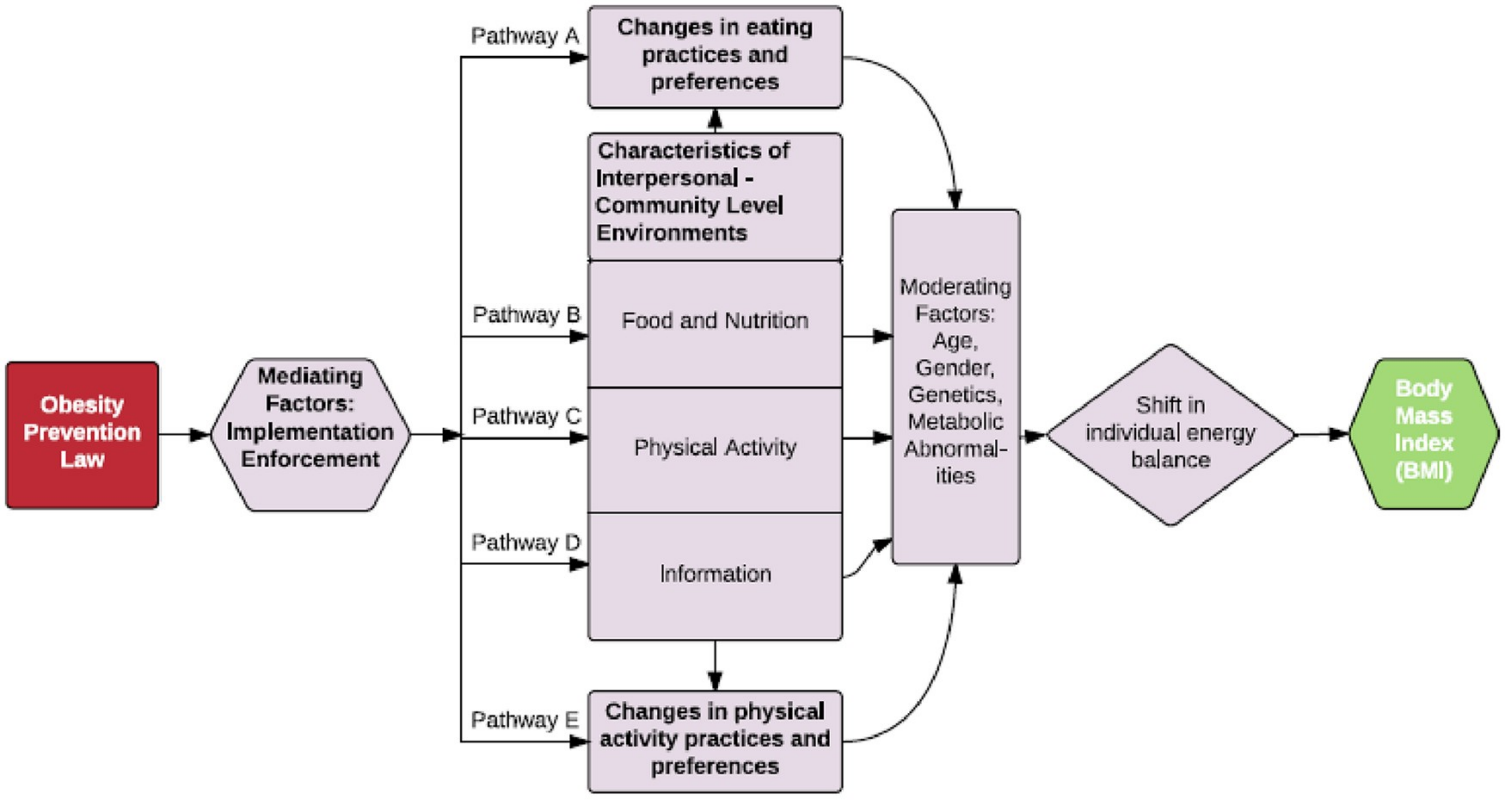
Conceptual model linking obesity prevention laws to body mass index (BMI)*. * Demographic factors are presented as moderators linking obesity prevention laws to BMI as they will affect the strength of the relationship between changes outlined in Pathways A-E and individual-level change in BMI; implementation and enforcement are mediators as the effect(s) of obesity prevention laws will depend upon how and to what extent they are experienced by the target population.

Building on this general framework, Fig 1 presents a conceptual model of the pathways through which state laws might bring about reductions in BMI. It combines the PHLR conceptual framework and the ecological model of health articulated in the literature on social determinants of health. The ecological model emphasizes the multiple effects of intrapersonal, interpersonal, organizational, community, and public policy factors on health [27, 28]. Ecological frameworks have been used to motivate an examination of the relationship between weight-related behaviors and individual and contextual determinants of health [29–40].

Sallis et al.[40] articulate four core principles of ecological models. First, health behaviors are influenced by factors at multiple levels. At the intrapersonal level, interventions focus on individual acquisition of knowledge, promoting a sense of self-efficacy, and changing health-related beliefs and expectations [40]. Increased knowledge of nutrition is associated with greater weight loss and more healthful food intake patterns [41, 42]. The interpersonal level focuses on an individual’s experience with family, work groups, peers, and social networks. An individual’s decisions about diet or physical activity may be influenced by their peer groups [43]. The organizational level refers to the organizational culture, norms, structure, and incentives that an individual experiences. Organizational settings can include daycare centers, schools or universities, workplaces, or churches and have the potential to exert significant influence on weight-related behaviors. A young person’s access to healthy foods or resources for physical activity may depend largely on the school environment [44].

Community ranges from a psychological sense of connectedness to groups of individuals with shared identities to individuals living in the same geographic location [45]. Neighborhood structure and resources are significant predictors of an individual’s physical activity levels and dietary patterns [46]. At the public policy or macroenvironmental level, health decisions are shaped- directly or indirectly- by local, state, and national statutes, regulations, and programs. An individual’s weight-related behaviors may be shaped by a state’s food tax laws, zoning regulations, or insurance policies. Researchers have also considered foundational, deeply-rooted aspects of contemporary society such as poverty and racial segregation as predictors of weight-related behaviors [47–49].

Second, influences on behavior interact across levels to shape health-related choices. Sallis and Owen suggest that individuals with high motivation to avoid weight gain (intrapersonal level) may react differently than those with lower motivation to driving past a fast-food restaurant (community level). Similarly, peer support for healthful eating (interpersonal level) may be more likely to change people’s eating habits when policies augment it with insurance coverage for nutrition counseling. Changes in law may operate at the policy level but have trickle-down effects at other levels.

Third, ecological models are behavior-specific. An ecological model for youth overweight or obesity should differ from an ecological model of underage alcohol consumption or smoking behavior in the mechanisms for change that it identifies at each level of influence. Fourth, intervening at multiple levels should be the most effective strategy to change behavior. The primary goal of ecological models is to inform the development of comprehensive intervention strategies that systematically target mechanisms of change at several different levels. While it is generally accepted that obesity in adults and children is the result of an individual’s positive energy balance over time, it is important to consider and respond to the many forces that create this balance by shaping eating and activity patterns [50].

Integrating these features of the ecological model of health with the PHLR framework suggests five different pathways along which an obesity prevention law might operate to change BMI (Fig 1). First, a law might target eating practices and preferences of an individual directly by, for example, providing individualized nutrition and wellness counseling (Pathway A). Second, a law could directly target a person’s physical activity behaviors and preferences (Pathway E). Both of these pathways operate at the intrapersonal level.

Pathways B, C, and D illustrate legal approaches that effect eating or physical activity indirectly by targeting components of the interpersonal, organizational, and community levels of influence on individual health behaviors. Laws that operate along Pathway B influence BMI by changing food environments that an individual experiences regularly. A law might require that schools limit the availability of vending machines during the school day. Pathway C laws alter dimensions of the environment that relate to physical activity, while Pathway D laws promote changes in the types of information that individuals are exposed to in different environments. For example, a law might require schools to eliminate advertisements for sugar-sweetened beverages.

Changes in each environment will have implications for individual eating and activity patterns. Over time, if these changes include eating smaller portions of food and engaging in more physical activity, an individual’s energy balance will shift from positive to negative, resulting in a decline in BMI. Moderating factors along the path from a change in eating and activity patterns to shift in energy balance include an individual’s age, gender, genetic predisposition to weight gain, and physiological and metabolic abnormalities (i.e., resting metabolic rate declines as energy intake decreases) [51, 52].

### Typology of obesity prevention laws

The foregoing conceptual framework suggests the classification system for OPLs in Table 1. Laws are categorized by ecological level of influence on weight-related behaviors and the legal mechanism utilized to change behavioral determinants of BMI. Laws may affect energy intake, energy expenditure, or the information environment. Energy intake may be altered through increased access to healthy foods or decreased access to obesogenic foods. Energy expenditure may be changed through increasing opportunities for physical activity or decreasing barriers to being more active. Finally, information about diet and exercise patterns conducive to maintaining a healthy weight may be made more readily available while information and messages about obesogenic foods could be minimized. We operationalized this conceptual framework and typology of laws to create a database of state OPLs adopted between 2000 and 2007, a period of intense legislative activity (S1 Table).

**Table 1:** Classification of obesity prevention laws by legal mechanism and ecological influence.

Mechanism		Ecological Level			
		Intrapersonal (individual)	Interpersonal (home)	Organizational (schools, daycares)	Community
**Energy Intake**	Increase access to healthy foods			Nutrition standards for school meals Nutrition standards for child care facilities Farm-to-school or school garden programs	Farmer’s market development Grocery store development
	Decrease access to obesogenic foods			Nutrition standards for competitive foods Nutrition standards for competitive beverages Restricted access to competitive foods or beverages	Ban on *trans* fat Snack or sugar-sweetened beverage tax
**Energy Expenditure**	Increase opportunities for physical activity			Physical activity standards for schools Physical activity standards for child care facilities Physical fitness assessments	Walking and bike paths
	Decrease barriers to physical activity				Safe routes to schools
**Information**	Increase access to health information	Private insurance coverage for wellness counseling Public insurance coverage for wellness counseling		Nutrition education curriculum standards Health education standards Physical education curriculum standards BMI reporting Diabetes screening School wellness policies	Menu labeling in restaurants
	Decrease exposure to obesogenic messages			Restricted advertising or marketing of competitive foods	

Gray shading indicates no laws were identified in these categories during the study period.

### Law search protocol

OPLs for all 50 states and DC were obtained via primary legal research in 2011 using the LexisNexis Advanced Legislative Services (ALS) database. The state was chosen as the unit of analysis because that has been the standard in PHLR in relation to the study of law or policy effects and because there has been substantial legislative activity in relation to obesity prevention at this level. State bills enacted between January 1, 2000 and January 1, 2008 were included in the database. Prior work suggested (and our results confirmed) that very few states had adopted OPLs prior to 2000. Keyword searches were developed to identify laws in the following areas: school food and nutrition, school physical activity and education, farm-to-school and school garden programs, childcare nutrition, childcare physical activity, school wellness policies, safe routes to school, body mass index monitoring and reporting, diabetes screening, grocery store and supermarket development, farmers’ market development, menu labeling requirements, advertising and marketing restrictions, taxes on sugar-sweetened beverages and snack foods, and public and private insurance coverage for nutrition and wellness counseling (S2 Table). We used an iterative, multi-stage procedure to create an exhaustive collection of OPLs [26]. The final list of categories is presented in Table 1. More detailed information about the search protocol is currently available at LawAtlas [53].

### Law coding protocol

We developed a detailed coding manual to guide the review and data extraction process.

A team of three reviewers examined 4199 state-level bills identified by running the final search terms in the ALS database. Each bill was coded by one reviewer. For each legal provision that met our inclusion criteria, reviewers abstracted information on the bill’s state, citation, passage and effective dates, target population, and category in our classification scheme. They also generated a narrative description of its purpose and intended effect. To determine effective dates, which vary by state, we applied the rules specified in the Effective Dates for State Legislation (EDSL) Chart developed by StateScape Policy Tracking and Analysis [54].

To ensure interrater reliability the reviewers independently coded the same random sample of 25 statutes and compared their results. This exercise revealed highly consistent agreement on the classification of a given law as well as narrative descriptions. To address some observed differences, exclusion criteria for walk and bike path laws were refined. S1 Table includes detailed information about inclusion and exclusion criteria. The complete coding manual and database codebook are available on LawAtlas or from the authors.

## Results

Table 2 displays the number and proportion of states with each of the 25 different types of OPLs in both 2000 and 2007. We identified 16 types designed to change the school (organizational) environment, 7 focused on community-level determinants of obesity, and 2 (laws requiring or encouraging public and private insurance coverage for health and wellness counseling) focused on the intrapersonal level.

**Table 2:** Prevalence of obesity prevention laws among U.S. States, 2000 and 2007^†^.

	States With the Provision			
	2000		2007	
Legal Provision Type	*n*	*%*	*n*	%
**Intrapersonal level interventions**				
**Private insurance coverage for wellness counseling**	2	4%	9	18%
**Public insurance coverage for wellness counseling**	--	--	7	14%
**Interpersonal level interventions**	0	0	0	0
**Organizational level interventions**				
**Farm-to-school or school garden programs**	--	--	11	22%
**Nutrition standards for school meals**	--	--	8	16%
**Nutrition standards for competitive foods**	--	--	17	34%
**Nutrition standards for competitive beverages**	--	--	17	34%
**Nutrition standards for childcare facilities**	1	2%	1	2%
**Restricted access to competitive foods**	--	--	1	2%
**Physical activity standards**	1	2%	48	96%
**Physical activity standards for childcare facilities**	--	--	1	2%
**Physical fitness assessments**	--	--	13	26%
**Health education standards**	1	2%	4	8%
**Physical education curriculum standards**	6	12%	40	80%
**Nutrition education standards**	2	4%	36	72%
**Restricted advertising or marketing of competitive foods**	--	--	3	6%
**School wellness policies**	--	--	9	18%
**Body mass index reporting**	--	--	5	10%
**Diabetes screening**	--	--	4	8%
**Community level interventions**				
**Farmer’s market development**	2	4%	14	28%
**Grocery store development**	--	--	2	4%
**Snack or sugar-sweetened beverage tax**	2	4%	12	24%
**Ban on *trans* fat**	--	--	--	--
**Walking and bike paths**	8	16%	37	74%
**Safe routes to schools**	--	--	7	14%
**Menu labeling in restaurants**	1	2%	5	10%

^†^ Unshaded rows represent interventions into the information environment. Light shading denotes interventions aimed at influencing energy intake. Darker shading denotes interventions aimed at influencing energy expenditure.

### Ecological levels of obesity law interventions

In 2000, few states had adopted OPLs at any ecological level. By 2007 there was tremendous growth of laws that targeted the organizational environment. Of the sixteen different organizational-level OPLs, only a handful—nutrition standards for child-care facilities, physical activity, physical education, health education, and nutrition education standards—were present in any state in 2000. By 2007, all sixteen were present in at least one state. Physical activity and physical education standards for schools went from virtually nonexistent to nearly universal (96% and 80% of states, respectively). While we observed the greatest growth during the study period in legal provisions that targeted the school environment, there was also substantial growth in community-level legal interventions—walk and bike path legislation was especially prolific, spreading from less than a fifth (16%) of states in 2000 to almost three-quarters (74%) by 2007.

### Legal mechanisms of obesity laws

The most commonly utilized legal mechanism was increasing opportunities for energy expenditure in the form of physical activity standards at schools (96% of states). Altering access to information about healthy behavior through physical education curriculum standards was a close second. Legal mechanisms designed to directly influence energy intake—for instance, through nutrition standards for school meals or competitive foods—were comparatively less common at only 16% and 34% of states, respectively. The greatest variation in the content of legal mechanisms was evenly split among those that sought to directly influence energy intake (10) and those that altered the information environment (10). Thus, while more states have successfully adopted laws that are designed to operate through influence on energy expenditure, there is greater experimentation in the realm of legal mechanisms designed to change dietary habits and access to health and nutrition information.

### Obesity prevention frameworks at the state level

The ecological model predicts that intervention strategies that target mechanisms of change at multiple ecological levels will be most successful. In order to examine the extent to which states were adopting a multilevel approach, we developed several illustrative packages of OPLs that would constitute a policy framework for obesity prevention. The most comprehensive package included at least one law that did each of the following: increased access to healthy foods, decreased access to obesogenic foods, increased opportunities for physical activity, and decreased barriers to physical activity at both the school and community level. An intermediate package consisted of at least one law that increased access to healthy foods and one law that decreased access to obesogenic foods at both the organizational and community level. The least comprehensive package included at least one law that increased access to healthy foods and at least one law that decreased access to obesogenic foods at the community level—for example, a farmer’s market law and a law taxing snacks or beverages.

No states had enacted the most comprehensive package by the end of 2007. California was the only state that had the intermediate framework, which included nutrition standards for school meals, restricted access to competitive foods and beverages in schools, grocery store development, and a tax on snacks or sugar-sweetened beverages.

## Conclusion

Public health law research and scholarship continues to focus on measuring the effects of a wide range of state laws on population health [55]. Recent studies have considered the effects of everything from medical marijuana legalization to seat belt safety laws [56–58]. While these studies and previous work on obesity prevention laws often make reference to the ecological model of health behavior change, they have not systematically linked this model to the laws under investigation. Moreover, the focus is generally on a particular ecological level, such as schools. In this paper, we presented a conceptual framework that allows for the classification of obesity laws based on the ecological level of influence and the underlying legal mechanism involved. This creates the capacity to develop theory-driven models of the effects of various combinations of legal techniques and environment modification. This is an important first step in the direction of identifying legal interventions that are most likely to reduce obesity prevalence and increase health-promoting behaviors.

Our classification system and search reveals that no obesity laws enacted during the study period specifically targeted the interpersonal level of influence. This is an important finding because it shows that a domain critical to shaping obesity-related behaviors and habits was not the target of substantial policy activity. Although changing dietary practices in the home is likely to be an essential component of any policy intervention that successfully reduces obesity rates over the long term, lawmakers may find it particularly challenging to develop politically feasible and socially acceptable interventions at this ecological level. Proposed amendments to the Supplemental Nutrition Assistance Program (SNAP) that would restrict the purchase of foods that do not meet certain nutritional standards provide an example of how a legal mechanism that decreases access to obesogenic foods might target the home environment indirectly [59]. Ultimately, efforts to quantify the effects of any current or proposed interventions must continue to evolve with a focus on linking laws to health outcomes with a solid theoretical model of health behavior change.

Our findings of extensive adoption of physical activity standards, physical education standards, and walk and bike path legislation are consistent with prior studies of the state-level obesity policy landscape [60–63]. Our finding that the majority of states do not have a comprehensive package of obesity prevention laws that targets multiple ecological levels of influence on youth obesity raises important questions about how obesity policy is developed on the state level as even more recent data collection efforts reveal that multilevel legal approaches within states are rare. Several studies [61–64] have sought to identify state-level socioeconomic, political, or population health factors that shape adoption of obesity prevention laws. Political factors such as a democratically controlled legislature and a longer legislative term are both associated with increased likelihood of bill enactment. State legislative action on obesity was also more likely in states with a greater gap between adults’ actual and desired weight, a higher percentage of college-educated adults, and a higher percentage of African-American residents [65]. However, adult obesity prevalence was generally not a significant predictor of bill enactment [65].

In order to create the potentially synergistic effects of multiple obesity prevention laws, state policymakers should continue to create and enact legal interventions that target different ecological levels of influence on youth obesity. Expanding opportunities for recreation through walk and bike path development is an important first step to encourage healthier lifestyle choices. However, it does not go far enough in addressing the multiple environmental determinants of obesity. There is a glaring lack of legal interventions that directly target the intrapersonal and interpersonal ecological levels; this highlights the need for laws that will change food and physical activities practices within the home and provide individuals with more opportunity to change their physical activity and food practices independently. For example, restricting the use of supplemental nutrition assistance benefits to healthy foods, providing benefits that cover the cost of using local gym facilities for families or individuals, or increasing individual access to obesity treatments through public or private insurance mechanisms are policy options that would start to fill the existing gap. Future research might examine the scope and content of the evidence base for successfully adopted obesity prevention laws in an effort to bridge the gap between obesity research and law. The absence of more comprehensive combinations of obesity prevention laws may limit the potential of legal interventions to help solve this pressing public health issue.

## Supporting information

S1 Table Criteria for assignment of obesity-related legal provisions to subcategories. (DOCX)

S2 Table Search terms for obesity-related legal provisions. (DOCX)
