# Supplementary material for: Multilevel legal approaches to obesity prevention: A conceptual and methodological toolkit
Source: PLoS One. 2019 Oct 1;14(10):e0220971. doi: 10.1371/journal.pone.0220971 (PMC6772030; doi:10.1371/journal.pone.0220971)
Supplement: S1 Table — (DOCX) [file pone.0220971.s001.docx]

**S1 Table: Criteria for Assignment of Obesity-Related Legal Provisions to Subcategories**

| **Subcategory** | **Code** | **General Description** | **Inclusion Criteria** | **Exclusion Criteria** | **Sample Legal Provisions** |
| --- | --- | --- | --- | --- | --- |
| **Major Category: Food & Nutrition** |  |  |  |  |  |
| Nutritional standards for school meals | NSM | States may set nutritional standards for school breakfasts, lunches and snacks that exceed the minimum requirements established by the USDA and are in accordance with the 2005 Dietary Guidelines issued by the Secretary of Agriculture. States may strength school nutrition standards by increasing the amount and variety of whole grain products, increasing the availability of fruits and vegetables for school meals, offering skim or low-fat milk, reducing sodium content in all meals, increasing fiber content, cutting cholesterol and minimizing or eliminating trans fats. | Policies that outline standards for foods or beverages sold as part of free, reduced-cost or full cost school meals, including restrictions on the sale of school meal items that do not meet specified nutritional guidelines, should be included. If the law or regulation also articulates standards for food or beverages sold outside of the school meal programs, it should be coded here and under "Nutritional standards for competitive foods and beverages". | N/A | *California SB 80: "This bill would require schools and school districts, commencing with the 2007-2098 fiscal year, for meals and food items sold and served as part of a free or reduced price meal program, to comply with specified nutrition-related prohibitions and requirements"* |
| Nutritional standards for competitive foods and beverages | NCF | Competitive foods and beverages are defined by the USDA as any foods and beverages that are sold at school but outside of the USDA school meals programs. Competitive foods and beverages include those items sold in vending machines, a la carte lines and school stores. States may choose to regulate the nutritional standards of these items. | Policies that outline nutritional standards for non-school meal program foods and beverages should be included. Policies that also include limitations on the availability of competitive foods and beverages should be coded here and under "Restricted access to competitive foods". | N/A | *2005 Colorado SB 81: "On or before July 1, 2006, each school district board of education is encouraged to adopt policies ensuring that every student has access to healthful food choices in appropriate portion sizes throughout the school day. At the minimum, this includes the provision of healthful items in vending machines, pursuant to Section 22-32-134."* |
| Restricted access to competitive foods | ACF | Limitations on when and where non-school meal program foods and beverages may be sold that go beyond federal requirements. | Policies that detail when and where competitive foods may be sold, including items from vending machines, school stores, bake sales, etc. | N/A | *2005 Maryland SB 473: "By August 1, 2006, all vending machines in public schools shall have and use a timing device to automatically prohibit or allow access to vending machines in accordance with the nutrition policy established and implemented by the county board."* |
| Farm-to-school programs and/or school gardens | FTS | Farm-to-school programs attempt to link local farmers with schools to increase the availability of fresh fruits and vegetables, reduce costs and support local business. The connection may be established administratively through farm-to-school program coordinators or by granting schools money to purchase locally grown produce. School garden programs attempt to increase availability of produce by creating student-run gardens. | Policies that are designed to connect local farmers with schools or increase schools' access to locally grown produce should be included in this category. "Schools" includes public or private institutions, colleges and universities. Any policy that encourages or requires schools to create opportunities for students to cultivate and consume fresh fruits and vegetables they have grown should also be included in this category. Policies that encourage or require the development of community-based gardens should not be coded here but placed in "Farmers' market development and community gardens". Commodity price support policies should not be included in this category. | Commodity price support policies should not be included.  Policies that encourage or require the development of community-based gardens should not be coded here, but rather, coded as FMG. | *2008 Oregon HB 3601: "The Department of Education shall establish the Oregon Farm-to-School and School Garden Program."* |
| Nutrition education standards | NES | These policies encourage or require more comprehensive instruction on nutrition and its relationship to overweight and obesity than existing health education courses. | Any policy that encourages or requires adoption of nutrition-focused curriculum should be included in this category. If the legislation or regulation in question also calls for physical education, it should also be coded under "Physical education curriculum standards". If the policy includes nutrition education as part of a broader school wellness program, it should be coded here and under "School Wellness Policies". | N/A | *2005 CA AB 689: "There is a need for both a sequential physical education that involves moderate to vigorous physical activity and teaches knowledge, motor skills, and positive attitudes and activities that all pupils can enjoy and pursue throughout their lives that are taught by well-prepared and well-supported staff, as well as health education content standards that incorporate nutrition and physical activity concepts, laying the foundation for lifelong healthy habit"* |
| School Wellness Policies | SWP | All schools districts that participate in the National School Lunch Program and/or School Breakfast Program were required to adopt and implement local school wellness policies by the 2004 Child Nutrition and WIC Reauthorization Act. School districts were required to establish nutritional guidelines for all foods sold on campus, ensure that federally reimbursable school meals meet minimum USDA standards, and establish goals for nutrition education, physical activity and other school-based activities. | Any policy enacted by the state legislature that requires or encourages the local school districts to implement federal guidelines for wellness policies should be included in this category. Any legislation that allocates funds or technical assistance to school districts to aid in the implementation of local school wellness policies should also be included in this category. Any policy that develops state-level oversight or review mechanisms for local school wellness policies should also be included in this category. | Policies that encourage or require health-related initiatives- including but not limited to adoption of nutritional standards for any type of foods or beverages sold at schools, nutrition education, physical activity or education standards, or farm-to-school or school garden programs should not be included in this category. | *2006 Florida SB 772: "BY SEPTEMBER 1, 2006, EACH SCHOOL DISTRICT SHALL SUBMIT TO THE DEPARTMENT OF EDUCATION A COPY OF ITS SCHOOL WELLNESS POLICY AS REQUIRED BY THE CHILD NUTRITION AND WIC REAUTHORIZATION ACT OF 2004...EACH SCHOOL DISTRICT SHALL ANNUALLY REVIEW ITS SCHOOL WELLNESS POLICY AND PHYSICAL EDUCATION POLICY AND PROVIDE A PROCEDURE FOR PUBLIC INPUT AND REVISIONS. IN ADDITION, EACH SCHOOL DISTRICT SHALL SEND AN UPDATED COPY OF ITS WELLNESS POLICY AND PHYSICAL EDUCATION POLICY TO THE DEPARTMENT WHEN A CHANGE OR REVISION IS MADE."* |
| Child-care licensing regulations - nutrition | CCN | A growing number of preschool aged children are overweight or obese and an increasing number spend some portion of the day in child care facilities. Child care facility standards attempt to improve the environment in which these children spend a significant amount of time through physical activity and nutrition standards, in addition to limitations on television consumption. | Any policy that requires or encourages adoption of nutritional standards for foods or beverages, limits the availability of foods of minimal nutritional value, or includes any other measure designed to improve the nutrition environment in child care facilities should be included in this category. "Child care facilities" refer to day care establishments of varying size that are responsible for the well-being of preschool aged children (birth up to 5 years of age in most cases). If the policy also presents the same set set of standards for school-aged children, it should also be coded in the appropriate categories that relate to schools. | Policies that relate only to physical activity standards should be coded instead under CCA | *Tennessee Department of Human Services, Licensure Rules for Child Care Centers, Chapter 1240.04.03.11(1)(b): Appropriate foods shall be encouraged; highly inappropriate foods, e.g. foods high in sugar and/or fat content, but containing low nutritional value, shall be discouraged.* |
| Advertising and marketing restrictions in schools | AMS | A report released by the IOM in December 2006 concluded that food and beverage marketing practices put children's long term health at risk. A subsequent Federal Trade Commission (FTC) report on the food and beverage industry's marketing to children and adolescents found that companies used a comprehensive range of mechanisms (television, radio, print, Internet) to promote their products and focused heavily on certain age groups depending on the product being sold. Advertising and marketing restrictions in school attempt to curb students' exposure to promotional messages for certain foods and beverages. | Any policy that requires or recommends limitations or a complete ban on the advertisement of foods and beverages on school grounds should be placed in this category. | Limited liability laws that grant immunity to marketers or advertisers against claims of weight gain associated with the advertised product. | *2007 Maine LD 184: "Brand-specific food or beverage advertising on school grounds is prohibited, except for water and product packaging."* |
| Grocery store and supermarket development | GSD | The presence of grocery stores and supermarkets is a predictor of how many fresh fruits and vegetables residents of certain areas consume. Grocery store and supermarket development initiatives usually strive to increase the number of full size grocery stores and supermarkets that serve low-income and rural populations. | Any policy that encourages or supports the expansion of existing convenience stores to provide healthier foods, or the introduction of new, full-service food retailers should be included in this category. | N/A | *2006 California AB 2384: The department, in consultation with the Department of Food and Agriculture, shall design the program to include the following two components: Strategies aimed at small grocers in targeted low-income neighborhoods to increase the offerings of fresh fruits and vegetables in those communities.* |
| Farmers' market development and community gardens | FMG | These policies are designed to increase access to fresh and nutritious foods, typically in areas with limited access to grocery stores, supermarkets and fresh produce. | Any policy that encourages government agencies (i.e., Department of Health, Department of Education, Department of Food and Agriculture) to purchase from local farmers, provides financial assistance to local farmers, or presents some mechanism to expand availability and access to farmers' markets or community gardens should be included in this category. If a policy also includes "farm-to-school" or "school garden" language, it should also be coded in that category. | N/A | 2008 South Carolina SB 1066: Creates and implements a statewide farmers' market system. |
| Menu labeling requirements for restaurants | MLR | Policies designed to help inform consumer choices by requiring nutritional information and content on the menus and menu boards at full service and fast food facilities. | Any policy that requires a restaurant to provide nutrition information, including but not limited to caloric content, nutritional composition, or fat grams per serving, on menu boards or menus , should be included in this category. "Restaurant" includes fast food restaurants, full service restaurants, chain and independent food facilities. | Policies that prohibit menu labeling. | 2008 California SB 1420: Restaurants with 20 or more locations in the state must disclose calorie and nutrition information in a "clear and conspicuous" manner. |
| Trans fat bans or nutritional  content  restrictions outside the school setting | TFB | Trans fat pose a threat to cardiovascular health and there is consensus among health officials that they should only be consumed in trace amounts. | These policies limit the amount or use of trans fat in foods prepared and sold outside of the home. | Policies that call for menu labels to contain information about trans fat content should not be placed in this category but rather, coded as MLR  Policies that limit or eliminate trans fat from foods sold in schools should not be placed here but rather, as NSM or NCF. | 2008 California AB 97: Prohibits oil, shortening, or margarine containing specified trans fats for specified purposes, from being stored, distributed, or served by, or used in the preparation of any food, commencing January 1, 2010. Also prohibits any food containing artificial trans fat, from being stored, distributed, or served by, or used in the preparation of any food within, a food facility, commencing January 1, 2011. |
| Snack and sugar-sweetened beverages tax | TAX | Financial incentives and disincentives have been considered by lawmakers as a means of encouraging healthy behaviors and discouraging unhealthy ones. Taxes on foods with minimal nutritional value, including certain snack foods and sugary drinks, is one approach. | Any policy that places a tax on certain items, at times deemed "junk food", "snack food", "sugar-sweetened beverages", "soft drinks" or "soda" should be included in this category. If the policy also includes limitations on the availability of these items, inside or outside of the school settings, it should also be coded in the appropriate category. | N/A | 1991 California AB 2181: This bill would eliminate as of July 1, 1991, total or partial exemptions from state and local sales and use taxes for (1) the gross receipts from the sale of and the storage, use, or other consumption in this state of candy or confectionery, nonmedicated chewing gum or snack foods, as defined. |
| Major Category: Physical Activity |  |  |  |  |  |
| Physical education standards | PES | Physical education (PE) standards refer to the quality and quantity of instruction that school-aged children receive. | Any policy that requires PE in schools, prohibits the elimination of PE, or encourages the expansion of PE offerings through allocation of money, technical assistance, human resources or other means should be included in this category. | Policies that encourage or require school districts to expand PE as part of local school wellness policies should not be included here, but rather, coded as SWP | 2008 Florida SB 610: Requires physical education in grades 6 through 8 beginning with the 2009-2010 school year at the equivalent of one class period per day of physical education for one semester each year. |
| Physical activity standards and recess legislation | PAS | Physical activity is an integral part of a healthy lifestyle. The 2008 Physical Activity Guidelines suggest that children and adolescents get one hour of physical activity each day. These standards are designed to increase the amount of activity that school-aged children receive. | Any policy that encourages or sets a minimum amount of time for activity during the school day, through exercise programs, fitness breaks, recess, classroom activity, or other similar means, should be included in this category. Any policy that requires local school boards to develop a plan for increasing or setting minimum standards for physical activity- independent of local school wellness policies- should also be included in this category. | Policies that mention physical activity standards as part of local school wellness policies. | 2006 Indiana SB 111: Beginning with the 2006-2007 school year, requires the governing body of each school corporation to provide daily physical activity for students in elementary school. The physical activity must be consistent with the curriculum and programs developed under IC 20-19-3-6 and may include the use of recess. |
| Physical fitness assessments | PFA | Policies designed to measure the fitness level of students at various stages during K-12 years; typically appear in conjunction with BMI reporting and diabetes screening. | Any policy that encourages or requires physical fitness testing for students should be included in this category. | N/A | 2005 South Carolina HB 3499: Among other provisions related to student health, nutrition, physical education and fitness, the law requires all K-12 schools in the state to participate in the South Carolina Physical Education Assessment and requires that fitness reports be sent home to parents in the 5th and 8th grades and high school. |
| Health education standards | HES | Health education refers to instruction on emotional, mental, physical and social dimensions of health, as well as personal health, family health, community health, injury prevention, etc. | Any policy that requires or recommends health education for school-aged children should be included in this category. If both physical education or physical activity and health education are mentioned in the same law or regulation, it should be placed in both categories. If nutrition education is mentioned as a component of health education that should be strengthened, then the law/regulation should be placed in health education alone. If nutrition education is mentioned or conceived of as a separate course from health education and mentioned in the same policy, the law or regulation should be coded in both categories. | Policies that pertain solely to physical education or activity or nutrition education should not be placed in this category but rather, as PES, PAS, or NES, as appropriate. |  |
| Safe routes to school | SRS | The US Department of Transportation offers grants through the federal Safe Routes to School Program to fund infrastructure improvements that encourage children to walk and/or bike to school. State legislatures and agencies may develop strategies for these funds that best suit the needs of school-aged children throughout the state. | Any policy that encourages or requires increased access to walking and bike paths en route to schools, through the allocation of funds to develop pathways, re-define crosswalks, hire crosswalk guards, build sidewalks, increase penalties for violating traffic rules, or other similar mechanisms, should be included in this category. | Policies that are designed solely to increase safe access to walk and bike paths for the general population and are not specific to schools should not be included in this category, but rather, coded as WBP. | 2005 New Jersey AB3602: Permits the construction of traffic calming measures on certain roads near schools. |
| Child-care licensing regulations – physical activity | CCA | A growing number of preschool aged children are overweight or obese and an increasing number spend some portion of the day in child care facilities. Child care facility standards attempt to improve the environment in which these children spend a significant amount of time through physical activity and nutrition standards, in addition to limitations on television consumption. | Any policy that requires or encourages physical activity or limits the quantity of media consumption over a certain period of time within licensed child care facilities, or includes any other measure designed to increase activity, should be included in this category. "Child care facilities" refer to day care establishments of varying size that are responsible for the well-being of preschool aged children (birth up to 5 years of age in most cases). If the policy also presents the same set set of standards for school-aged children, it should also be coded in the appropriate categories that relate to schools. | Policies that relate only to nutritional standards should be coded under CCN | *Tennessee Department of Human Services, Licensure Rules for Child Care Centers, Chapter 1240.04.03.09(1)(c): There shall be alternating periods of vigorous activity and quiet play or rest throughout the day.* |
| Access to safe walking and bike paths | WBP | Various infrastructure development and community design measures that increase opportunities for safe physical activity in the form of walking and biking in urban or rural areas. | Any policy that encourages or requires increased access to walking and bike paths, through the allocation of funds to develop pathways, re-define crosswalks, hire crosswalk guards, build sidewalks, increase penalties for violating traffic rules, or other similar mechanisms, should be included in this category. | Policies that designed solely to increase safe access to schools should not be included in this category, but rather, coded as SRS. | 2008 California AB2971: Sets forth the Fair Share for Safety Program. Regarding the apportionment of federal transportation safety funds, to the extent possible, shall fund projects that provide safety benefits to both bicycle and pedestrian travel. |
| Major Category: Healthcare, Screening & Medical Intervention |  |  |  |  |  |
| Body Mass Index (BMI) monitoring and reporting | BMI | States may require school entry health certificates or student reports to include information about a student's body mass index. The BMI information may be used to identify youth at risk of developing Type 2 diabetes. | Any policy that requires the collection and report of student BMI information,individually or in aggregate, should be included in this category. Any policy that also calls for student health or fitness assessment that goes beyond BMI score should also be placed in "Physical fitness assessments", "diabetes screening and management", or any other relevant category. | N/A | *2007 Arkansas HB 1173: "Beginning with kindergarten and then in even numbered grades, require schools to includes as a part of a student health report to parents an annual body mass index percentile by age for each student."* |
| Diabetes screening | DBS | Type 2 diabetes has become increasingly common in adolescents and young adults, primarily due to the rise in overweight and obesity in this population. These policies are designed to identify at-risk members of the target population. | Any policy that recommends or requires diabetes screening among students- typically through a noninvasive procedure- should be included in this category. Type 2 diabetes education campaigns should also be included. Any policy that also calls for BMI reporting or fitness assessments should also be placed in the appropriate category. | Policies that call for the development of student diabetes care procedures and/or facilities on school campuses  Policies that allow school personnel to deliver care to diabetic students who suffer complications during the school day with limited liability  Policies that pertain to insurance coverage and treatment of Type 2 diabetes | 2007 Texas SB 415:Establishes a student risk assessment program for type 2 diabetes in certain regions of the state that includes screening of body mass index for students identified by a noninvasive screening as at risk for type 2 diabetes. |
| Private insurance coverage for nutrition and wellness counseling | PRI | States may recommend or require that health insurers operating in the state provide a number of different benefits, including nutrition counseling, weight loss and weight management programs, that might help reduce obesity rates. | Any policy that requires or recommends that private health insurers offering plans within the state provide obesity prevention benefits, such as nutrition counseling, wellness services, weight loss programs, weight management programs or similar services, should be included in this category. | Policies that only mandate insurance coverage of gastric bypass surgery as a treatment for obesity. | N/A |
| Public insurance coverage for nutrition and wellness counseling | PUI | State insurance programs for low-income and disabled residents may provide a number of different benefits, including nutrition counseling, weight loss and weight management programs, that might help reduce obesity rates. | Any state Medicaid program that provides obesity prevention benefits, such as nutrition counseling, wellness services, weight loss programs, weight management programs or similar services, should be included in this category. | Policies that only mandate public insurance coverage of gastric bypass surgery as a treatment for obesity | 2005 Colorado HB1066: Requires the department of health care policy and financing to develop and implement an obesity treatment program for the purpose of treating a Medicaid recipient who has a body mass index that is equal to or greater than 30 and who has a comorbidity related to the recipient's obesity including but not limited to diabetes, hypertension, and coronary heart disease. |
