# Supplementary material for: Multilevel legal approaches to obesity prevention: A conceptual and methodological toolkit
Source: PLoS One. 2019 Oct 1;14(10):e0220971. doi: 10.1371/journal.pone.0220971 (PMC6772030; doi:10.1371/journal.pone.0220971)
Supplement: S2 Table — (DOCX) [file pone.0220971.s002.docx]

**S2 Table: Search Terms for Obesity-Related Legal Provisions**

| **OPL Subcategories** | **Search Terms Used to Identify Laws** |
| --- | --- |
| Nutritional standards for school meals  Nutritional standards for competitive foods and beverages  Restricted access to competitive foods  Increasing and promoting access to fresh  produce   - Farm-to-school programs and school gardens   Nutritional education curriculum standards  Relevant childcare licensing regulations  Advertising and marketing restrictions | Nutrition, food, diet, snack, meal, lunch, calorie, beverage, soft drink, soda, vending, farm, garden, advertise, nutritional value, school, education, curriculum, children, pupil, student, teen, kid, youth, infant, toddler |
| School wellness policies | wellness, health education, healthy, lifestyle, school, active, school wellness policy, SWP |
| Access to healthy food   - Grocery store and supermarket development - Farmers’ market development | Farmers’ market, farm to school, school garden, senior, fruit , vegetable , agriculture, school , grocery , supermarket , education, fresh , school wellness policy , SWP, community , neighborhood , program , vendor |
| Menu labeling requirements for restaurants  Trans fat bans or nutritional content standards | nutrition information , menu , calorie , food facility , trans fat |
| Taxes on snack foods and sugar-sweetened beverages  Advertising and marketing restrictions | Tax, snack, junk , nutritional value, food |
| Taxes on snack foods and sugar-sweetened beverages | Tax, soft drink, sugar, sweet, beverage, soda |
| Physical education requirements  Physical activity standards  Physical fitness assessments  Health and physical education  Recess legislation  Safe routes to school  Relevant child-care licensing regulations | Physical activity, physical education, fitness, fitness assessment, recess, obesity , overweight , safe routes to school , SRTS, curriculum , children , pupil , student |
| Walk & bike paths | Walk, pedestrian , bike , bicycle, lane, path, trail , footpath  , greenway , complete streets, health, lifestyle , activity |
| Body Mass Index (BMI) monitoring and reporting | body mass index |
| Diabetes screening | Diabetes screening, diabetes prevention, diabetes education |
| Medicaid coverage for nutrition and wellness counseling  Private insurance coverage for nutrition and wellness counseling | Medicaid , State Childrens Health Insurance Program , SCHIP , health insurance, obesity , obese , overweight , body mass index , BMI , wellness |
